# Erectile dysfunction in cardiovascular patients: A prospective study of the *eNOS* gene T‐786C, G894T, and INTRON variable number of the tandem repeat functional interaction

**DOI:** 10.1111/andr.13671

**Published:** 2024-07-01

**Authors:** Ana Segura, Javier Muriel, Pau Miró, Laura Agulló, Vicente Arrarte, Patricia Carracedo, Thomas Zandonai, Ana M Peiró

**Affiliations:** ^1^ Urology Department, Andrology Unit Dr. Balmis General University Hospital Alicante Spain; ^2^ Clinical Pharmacology Department Pharmacogenetic Unit Dr. Balmis General University Hospital, Alicante Institute for Health and Biomedical Research (ISABIAL) Alicante Spain; ^3^ Department of Applied Statistics and Operational Research, and Quality Universitat Politecnica de València, Campus of Alcoi Plaza Ferrandiz y Carbonell s/n Alcoy Spain; ^4^ Cardiology Department Dr. Balmis General University Hospital Alicante Spain; ^5^ Department of Statistics and Applied Operational Research and Quality Universitat Politècnica de València Valencia Spain; ^6^ Department of Psychology of Developmental and Socialization Processes “Sapienza” University of Rome Rome Italy; ^7^ Clinical Pharmacology, Toxicology and Chemical Safety Unit Institute of Bioengineering Miguel Hernández University Avda. de la Universidad s/n Elche Spain

**Keywords:** cardiovascular diseases, eNOS gene, erectile dysfunction, pharmacogenetics

## Abstract

**Background:**

Cardiovascular disease induces erectile dysfunction modulated by endothelial nitric oxide synthase enzyme and an impaired ejection fraction that restricts penis vascular congestion. However, the mechanisms regulating endothelial dysfunction are not understood.

**Objectives:**

Exploring the functional impact of endothelial nitric oxide synthase genetic polymorphisms on erectile dysfunction and drug therapy optimization in high‐risk cardiovascular disease patients.

**Materials and methods:**

Patients with erectile dysfunction symptoms and candidates for andrology therapy were included (*n* = 112). Clinical data and endothelial nitric oxide synthase rs1799983 (G894T) and rs2070744 (T‐786C), genotyped by fluorescence polarization assays, were registered. The 27‐bp variable number of the tandem repeat polymorphism in intron 4 (intron4b/a) was analyzed by polymerase chain reaction‐restriction fragment length polymorphism. Association analyses were run with the R‐3.2.0 software.

**Results:**

A significant association between endothelial nitric oxide synthase 786‐TT (*p* = 0.005) and the aa/ac of intron 4 variable number of the tandem repeat (*p* = 0.02) with higher erectile dysfunction susceptibility was observed in cardiovascular disease patients (60 ± 9 years, 66% severe erectile dysfunction, 56% ejection fraction). After 3‐months of phosphodiesterase type 5 inhibitors, erectile dysfunction (International Index of Erectile Function, 50 ± 16 scores, the International Index of Erectile Function‐Erectile Function 21 ± 10 scores, *p* < 0.001) and sexual quality of life (modified Sexual Life Quality Questionnaire 55 ± 23 scores, *p* < 0.001) had significantly improved. The cardiovascular ejection fraction was influenced positively with better sexual quality of life (0.1941), and also in the endothelial nitric oxide synthase G894‐T allele (*p* = 0.076) carriers, which could merit future analyses. Erectile dysfunction was present as the primary clinical manifestation in 62% of cases, with cardiovascular disease occurring concurrently. Only former smokers and obese subjects debuted prior to cardiovascular disease than to erectile dysfunction.

**Conclusions:**

Our study provides comprehensive insights into the functional interaction linking endothelial nitric oxide synthase gene polymorphisms, erectile function, and ejection fraction in high‐risk cardiovascular disease patients. Future therapeutic strategies could target endothelial nitric oxide synthase activity by including lifestyle changes and epigenetic modulations.

## 1 INTRODUCTION

Cardiovascular disease (CVD) has been recognized as the commonest cause of erectile dysfunction (ED), whose importance as a sentinel symptom has grown.^1^ Both share a similar endothelial dysfunction pathophysiology related to age,^2^, ^3^ hypertension, smoking, or diabetes.^4^, ^5^ Different studies have found that patients with moderate or severe impairments of ejection fraction had significantly increased ED.^6^, ^7^ Thus, identification of the biomarkers that could accurately predict particular risk phenotypes may help cardiovascular preventive medicine.^8^ Even more because more than one‐third of ED patients ignore their underlying health problem^9^ with potential future cardiovascular consequences.

Nitric oxide (NO) is an important protective molecule in the vasculature, and endothelial NO synthase (eNOS) is responsible for most produced vascular NO^10^ that is proposed to regulate coronary blood flow and cardiac performance.^11^, ^12^, ^13^ eNOS has been one of the most studied candidate genes in penile corpus cavernosum smooth muscle pathway relaxation and for mediating vasodilatation^14^, ^15^, ^16^, ^17^ as the T786C and intron 4 (4b/a).^18^ The individuals carrying the 894‐T allele generate low NO in vivo and may be more susceptible to endothelial dysfunction, which might account for higher ED risk.^19^ Lower eNOS mRNA and serum nitrite/nitrate levels have been found in individuals homozygous for the C allele of the T786C polymorphism.^20^ There is also the 4b/4a variable number of the tandem repeat (VNTR) polymorphism in intron 4 which is able to regulate eNOS post‐transcriptionally by altering the formation of small interfering RNA (siRNA) and by, consequently, lowering eNOS mRNA levels in five (variant 4b) versus four (variant 4a) copy^8^, ^21^ variants. Thameem et al.^22^ showed that the patients carrying 27 bp‐VNTR exhibited a nominally significant association with cardiovascular measures after adjusting for trait‐specific covariate effects. However, the results of most association studies are presently inconsistent.^23^

The present study aimed to explore the functional impact of eNOS genetic polymorphisms in high‐risk CVD and ED patients. This approach will provide a better understanding of the role of eNOS genetic variants in endothelial dysfunction pathophysiology.

## 2 MATERIALS AND METHODS

### 2.1 Study population

A real‐world prospective observational study was conducted over a 3.5‐year period, from January 2018 to January 2023, except for 18 months (from December 2019 to June 2020) due to the coronavirus disease 2019 pandemic. Patients were recruited following their routine clinical visits for a Cardiac Rehabilitation Programme (Health Department of the Alicante General Hospital, Spain), which attends to 350 patients per year. In all, 155 patients out of 980 patients (15%) were referred to the Andrology Unit. Forty‐three subjects (3%) at follow‐up were missing, mostly due to not being willing to participate or to continue in the study, or for not meeting the full inclusion criteria for having ED.

At the time of enrolment, all the participants received information on the design and purpose of the study and provided their written informed consent to allow their genetic samples and electronic health records (EHRs) to be used for research purposes. All the methods were carried out in accordance with the ethical guidelines set out in the Declaration of Helsinki. The Research Ethics Committee of the Alicante General Hospital approved the protocol (code: PI2017/03), which complied with applicable STROBE guidelines.

Professional nurses interviewed all those who participated in cardiovascular rehabilitation programs during a standardized interview using a structured questionnaire that covered socio‐demographic characteristics and lifestyle factors (including physical activities, cigarette, and alcohol use). Total cigarette consumption (pack‐years) was estimated by taking daily consumption, multiplied by consumption years. A health professional also completed a medical details questionnaire. All the patients received a thorough regular medical examination, including medical and drug histories.

Following the questionnaire data collection, the patients who mentioned any sexual disorder were referred to the Andrology Unit for diagnosis and treatment. The referred patients were followed up for a 3‐month period upon treatment. Patients’ age ranges were 18−80 years old, and none had been taking hormone medication (supplementation or deprivation) or any phosphodiesterase type 5 (iPDE5) inhibitors before being recruited in the present study. Patients were asked about the sexual disorder that they had experienced prior to CVD onset.

### 2.2 Cardiovascular outcomes

High‐risk CVD individuals were included if they presented myocardial infarction or angina, and had been positively diagnosed with CVD. Individuals were excluded if they had been previously diagnosed with spinal cord or pelvic nerves (e.g., multiple sclerosis, multisystem atrophy, spinal cord injury, and tumors), and any conditions that affected the cauda equine, such as prolapsed intervertebral discs or tumors, disease to the parasympathetic nerves within the pelvis, individuals having undergone extensive surgery to the pelvis or abdomen, and conditions like chronic renal failure, hyperprolactinemia, smooth muscle dysfunction, and Peyronie's disease.

### 2.3 EF outcomes

The International Index of Erectile Function (IIEF) questionnaire was used to assess male sexual function, particularly the presence or absence of ED. The IIEF consists of 15 items grouped into five sexual function domains: EF, orgasmic function, sexual desire and satisfaction with sexual intercourse, and overall satisfaction. Higher scores for this questionnaire correspond to lower degrees of dysfunction. The domain assessing EF (IIEF‐EF) includes six questions (maximum score of 30) and is a reliable measure for classifying the degree of ED as severe (1–10), moderate (11–16), mild (17–25), and normal/no dysfunction (≥26 scores).^24^ Patients were also asked to complete a questionnaire, the modified Sexual Life Quality Questionnaire (mSLQQ‐QOL), on sexual quality of life. The mSLQQ‐QOL is a multidimensional tool with which patients and their partners are asked to compare their experiences prior to ED onset to their experiences since treatment began. It presents 10 assessment items: frequency of sex; duration of sex; ease of insertion; ease of achieving orgasm; ease of initiating sex; pleasure of anticipation; carefree feelings during sex; pleasure of orgasm; pleasure overall; partner pleasure.^25^ Prescriptions were made as usual for ED management and included iPDE5 (sildenafil, tadalafil, avanafil, or vardenafil).

To measure the impact of mental health, Hospital Anxiety and Depression Scale (HADS) questionnaire scores were used. There are seven items each for the depression and anxiety subscales. Scoring for each item ranges from 0 (no impairment) to 3 (severe impairment). A total subscale score of > 8 points out of a possible 21 denotes considerable anxiety or depression symptoms.

### 2.4 eNOS genotyping

DNA was extracted from 2 mL of saliva collected in phosphate‐buffered saline (PBS) buffer‐containing tubes. DNA quality control was photometrically verified. The determination of nucleotide changes was made by the polymerase chain reaction‐restriction fragment length polymorphism (PCR–RFLP) technique using isolated genomic DNA. Genomic DNA was isolated with the E.N.Z.A. Forensic DNA Kit (Omega bio‐tek) according to the manufacturer's instructions. The genotyping of the T786C (rs2070744) and G894T (rs1799983) eNOS polymorphisms was performed by a real‐time PCR (RT‐PCR) using specific TaqMan probes MGB (Applied Biosystems). All the PCR amplifications were done in an RT‐PCR Rotor Gene Q (Qiagen). The amplification parameters were as follows: 10 min initial denaturation at 95°C, 40 cycles for 15 s at 92°C, 90 s at 60°C, and 1 min final extension at 60°C. For 27‐bp intron 4 VNTR eNOS polymorphism genotyping, conventional PCR was performed with 200 ng of DNA. There were three different alleles for this polymorphism: allele “a” of 453 bp, “b” of 480 bp, and “c” of 507 bp. The amplification parameters were as follows: 15 min initial denaturation at 96°C, 35 cycles for 30 s at 95°C, 20 s at 59°C, 45 s at 72°C and a 10‐minute final extension at 72°C. The primers used for PCR amplification were: forward 5′‐ TGGAAAGGTAGGGGGACTG‐3′ and reverse 5′‐ GGTCACAGGCGTTCCAGTA‐3′. PCR products were loaded in 4% agarose gel and visualized under ultraviolet light.

### 2.5 Statistical analyses

Quantitative data are presented as the mean ± standard deviation (SD). The Shapiro‐Wilk test was used to test for normality. The comparisons for the continuous or categorical data between the two groups were made by an independent t‐test or Chi‐square test, respectively.

The statistical analysis of the clinical, biochemical, and socio‐demographical characteristics was performed using R 3.2.0. The Epi InfoTM v.7 software was utilized to compare the distribution of alleles, genotypes, and other categorical variables. Fisher exact probability was employed to test for associations. The multiple comparison *p*‐values were corrected by Bonferroni correction. An analysis of haplotypes was carried out by the SNPStats web tool. A probability of a null hypothesis lower than 5% was considered significant.

The relative frequencies of genotypes and alleles were calculated for each group. A chi‐square analysis was conducted to compare the distribution of genotypes and alleles. As there were only a few homozygotes per polymorphism, subjects were also grouped as carriers and non‐carriers for the analyses; that is, those who tested positive for the presence of allelic variants (dominant model) were defined as participants. The association between IIEF‐EF and improved EF upon treatment and the genotyped SNPs were examined by multiple logistic regressions. The associations between ED and CVD (ejective fraction) were examined with multiple logistic regressions. A *p*‐value < 0.05 was considered statistically significant. Analyses were carried out with the R software package, version 3.2.4.

## 3 RESULTS

**FIGURE 1:**
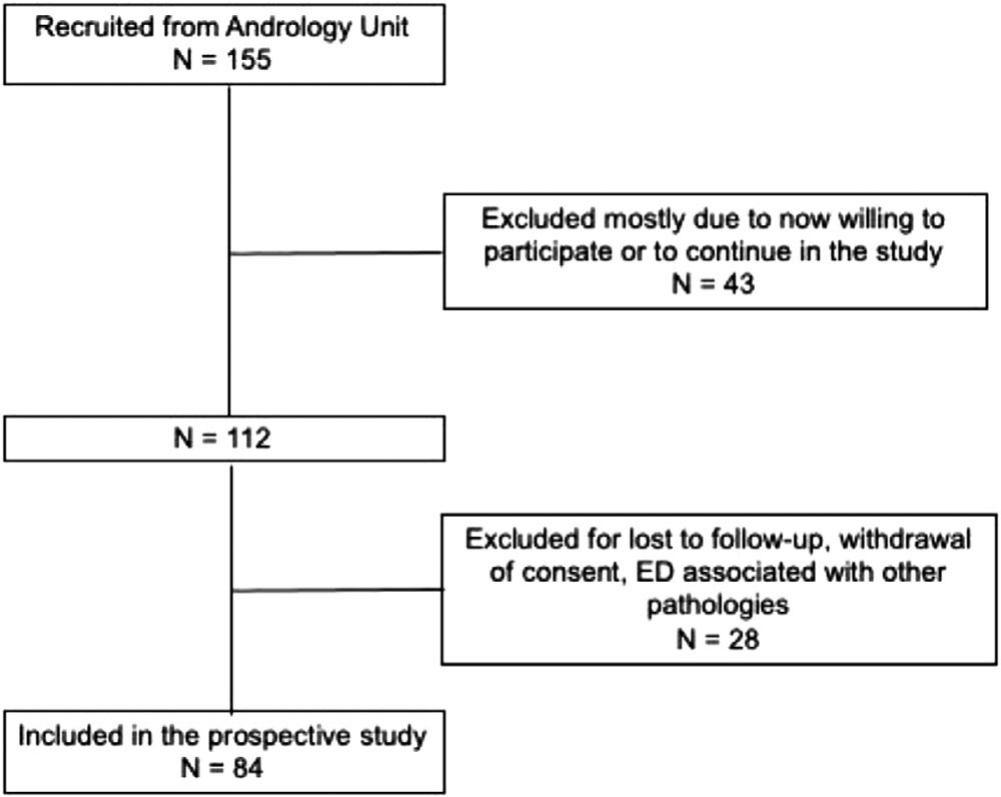
Flow chart of study inclusion presenting the recruitment of the subjects for a 3.5‐year inclusion period. Patients were referred from the Cardiovascular Rehabilitation Programme to the Andrology Unit (*n* = 155) for erectile dysfunction (ED) screening and management.

All the 155 CVD participants who reported decreased libido and/or ED were referred to the Andrology Unit, where ED was confirmed in 112 cases. Thus, a total of 25% (28/112) of the patients were lost to follow‐up due to withdrawing consent, ED associated with other pathologies, among others. In the end, 84 male Spanish CVD patients with ED finished the prospective study (Figure 1).

### 3.1 Study population

At the baseline, the CVD patients (*n* = 112, 61 ± 9 years, 100% male Caucasian Spaniards, body mass index [BMI] 29 ± 5 kg/m^2^) presented a normal ejection fraction (56%). The average CVD duration was 7 ± 67 years, and participants were treated mostly with statins (87%), aspirin (85%), and/or beta‐blockers (72%). Here 66% showed severe ED and a mean poor sexual quality of life (27 + 3 scores). The demographic and clinical variables of those included are shown in Table 1.

**TABLE 1:** Socio‐demographic and biochemical characteristics of the total population (*n* = 112 men) with erectile dysfunction and cardiovascular disease during the baseline visit to the Andrology Unit.

Basal variables	Mean ± SD
**Age** (years)	60 ± 9
**IIEF‐EF severe** (< 10 scores %),	66
**Cardiovascular ejection fraction** (%)	56
**Age upon first episode (**years)	
Erectile dysfunction	56 ± 10
Cardiovascular disease	54 ± 10
**Laboratory test** (mg/dL)	
Total cholesterol	136 ± 36
HDL cholesterol	42 ± 12
Triglycerides ±	142 ± 77
Creatinine	1.8 ± 6.2
**Vascular risk factors (%)**	
Hypertension	70
Dyslipidaemia	70
Diabetes mellitus	53
Former smoker	29
Obesity	34
Metabolic syndrome	31

Abbreviation: IIEF‐EF, Index of Erectile Function Questionnaire (erectile function).

Middle‐aged and elderly male participants were mainly married (77%), followed by divorced (13%), single or widowed (10%), and retired (43%), followed by those who presently work (37%). Hypertension and dyslipidemia were present in 70% of the population. Diabetes, obesity, or metabolic syndrome were observed in 33%–47%, while 49% of the total population included 28%, former smokers.

### 3.2 Temporal relation of ED and CVD

The data showed that 50% of individuals had experienced ED first, 38% had experienced CVD first, and 12% had noted both conditions at the same age. There were no significant differences in the baseline characteristics, cardiovascular risks, and lipid values, except for a significantly higher percentage of former smokers (12% vs. 38%, *p* = 0.002) and obese participants (23% vs. 41%, *p* = 0.062 with a marginal significance) prior to CVD debut than to ED, as Table 2 shows.

**TABLE 2:** Descriptive data when comparing patients debuting with erectile dysfunction (ED) and/or cardiovascular disease (CVD) upon inclusion. Significant differences (*p* < 0.05) are depicted in bold.

Variable, mean (SD) at baseline	ED first (*n* = 56)	CVD first (*n* = 42)	*p*‐Value
**BMI (kg/m^2^)**	28 ± 4	29 ± 5	0.146
**Ejection fraction (%)**	54	58	0.107
**Laboratory test (mg/dL)**			
**Total cholesterol**	142 ± 45	131 ± 27	0.300
**HDL cholesterol**	42 ± 10	43 ± 13	0.810
**Triglycerides**	144 ± 70	141 ± 8	0.456
**Creatinine**	2.5 ± 9.4	1.2 ± 1.1	0.563
**Vascular risk factors (%)**			
Hypertension	29	41	0.799
Diabetes mellitus	23	30	0.829
Dyslipidaemia	30	39	0.756
**Former smoker**	5	23	0.002*
Obesity	10	23	**0.062**
Metabolic syndrome	12	19	0.303
**Clinical outcomes**			
**IIEF** (5–75 scores)	32 ± 12)	34 ± 13	0.680
**IIEF‐EF** (1–30 scores)	9 ± 5)	10 ± 6	0.254
**mSLQQ‐QOL** (0–100 scores)	27 ± 11)	27 ± 27	0.663
**HADA scale** (0–21 scores)	6 ± 5)	6 ± 4	0.907
**HADD scale** (0–21 scores)	4 ± 5)	4 ± 4	0.690
**Pharmacology management (%)**			
ACE inhibitors	30	35	0.7752
Aldosterone	4	5	0.9999
Alpha blocker 1	2	11	0.1629
Antidepressants	2	11	0.1629
Antithrombotic agents	13	10	0.7872
ARA‐II	33	38	0.6981
Aspirin	89	84	0.6402
Beta‐blockers	67	76	0.4244
Calcium channel blockers	22	24	0.9814
Diuretics	11	29	0.0456
Ezethimide	35	22	0.2176
Hypoglycaemic agents	41	40	0.9999
Insulin	15	17	0.9599
iPDE5	80	71	0.3947
Statins	83	89	0.5102
Thienopyridines	24	32	0.4963

Abbreviations: ACE inhibitors, angiotensin‐converting enzyme inhibitors; ARA‐II, angiotensin II receptor antagonists; BMI, body mass index; HDL, high‐density lipoprotein; Hospital Anxiety and Depression Scale (HAD) as Anxiety (HADA) or Depression (HADD),IIEF‐EF, Index of Erectile Function questionnaire (erectile function); iPDE5, phosphodiesterase type 5 inhibitors; mSLQQ‐QOL, the modified Sexual Life Quality Questionnaire.

**FIGURE 2:**
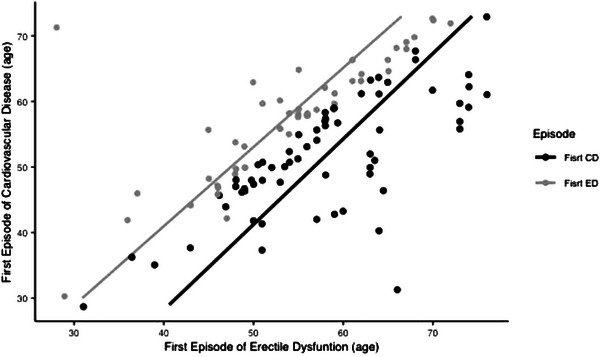
Correlation of age (years old) upon onset of erectile dysfunction (ED) and cardiovascular disease, using real‐world observational data in the Andrology Unit (*n* = 112).

As shown in Figure 2, there was a correlation between the age at first onset of ED and CVD as life span increases. Thus a significant increase in ED and CVD with age was observed. However, the correlation was strong (R^2^ 0.88) in the group of ED debut first, which is suggested to be a good predictor of CVD. In the other group (CVD debut), the relation correlated less (R^2^ 0.62) with more variability between onsets. Concomitant ED and CVD accounted for 12%. As this was not part of our objectives (ED as a sentinel symptom), we decided to not include this in Figure 1 in an attempt to reduce variability.

### 3.3 Contribution of the eNOS genotypes to ED in CVD patients

The genotype and allele frequencies for the T786C and G894T polymorphisms agree with the Hardy‐Weinberg Equilibrium. Table 3 shows that the frequency of the wild‐type form of the T786C and G894T genotypes was 20% and 46%, respectively, with similar Caucasian prevalence. Intron 4 VNTR was studied as previously described, where allele was (a) present in 16%. A comparison between the allele frequency in our study and the data from 1000 genomes was done by the Mann‐Whitney U test.

**TABLE 3:** Allelic frequencies of the T786C, G894T genotypes, and intron 4 variable number of the tandem repeat (VNTR) of the endothelial nitric oxide synthase (eNOS) gene when comparing our study frequency to other populations. Results of 106 genotyped patients.

Polymorphism	Genotype, *n* (%)			Wild‐type frequency	
				Study (*n* = 112)	Population (*n* = 1006)
**T786C (rs2070744)**	21 (20) (TT)	57 (54) (CT)	28 (26) (CC)	47% (T)	56% (T)
**G894T (rs1799983)**	47 (46) (GG)	53 (51) (GT)	3 (3) (TT)	71% (G)	66% (G)
**Intron 4 VNTR**	17 (16) (aa/ac)	89 (84) (bb/bc)	–	9% (a)	2% (a)

**FIGURE 3:**
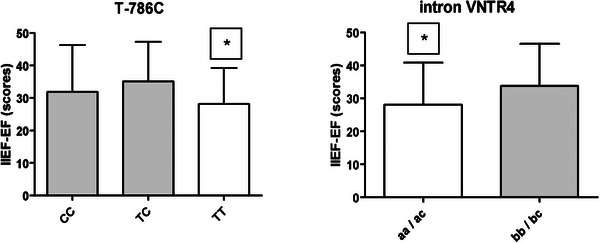
The International Index of Erectile Function (IIEF) questionnaire values during the Andrology Unit baseline visit due to the T‐786C (*p* > 0.001) and intron VNTR4 *eNOS* (*p* < 0.001) genotypes.

**FIGURE 4:**
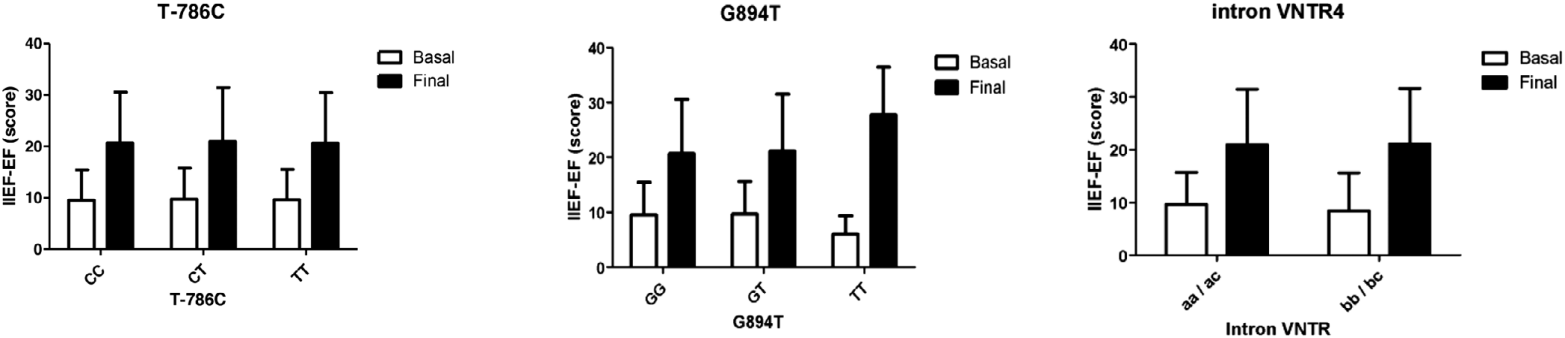
Functional impact of *eNOS* genetic polymorphisms on erectile dysfunction (ED) and drug therapy optimization in high‐risk CVD patients through the International Index of Erectile Function (IIEF‐EF) questionnaire during the basal and final visits to the Andrology Unit.

At the baseline, the patients carrying the eNOS wild‐type 786‐TT genotype and the aa/ac genotypes of intron 4 VNTR presented significantly worse ED when they were recruited, as shown in Figure 2. Three months after andrological treatment, the CVD patients reported significant ED improvement, as shown in Table 4. The patients treated with iPDE5 evidenced a significant improvement in EF, with increases of 66% for IIEF (50 ± 16 scores), 47% for IIEF‐EF (21 ± 10 scores, *p* < 0.001) and 50% for sexual quality of life (mSLQQ 55 ± 23 scores, *p* < 0.001). Anxiety and depression levels obtained scores lower than 8, which denote no mental health impairment within the same pre‐ and post‐range andrological treatments. However, a significant score degree was observed during the final visit. Pharmacological management appears in Table S1. Here the most used drugs were aspirin (86%), statins (86%), and beta‐blockers (72%), but with no significant difference between the ED or CVD first groups (see Table 2). We further analyzed the interaction of eNOS polymorphisms in relation to the ED response to iPDE5 management. As seen in Figure 3, after 3 months of andrological treatment genotypes did not significantly condition a different EF (before and after adjusting for age or BMI) with the IIEF score, regardless of whether the subject debuted with ED or with CVD. In contrast, a correlation analysis showed that the cardiovascular ejection fraction was not influenced by IIEF (−0.058), IIEF‐FE (−0.061), and MSLQQ‐QOL (0.194). The G894T polymorphisms had a slight influence (*p* = 0.076) on the distribution of the ejection fraction between genotypes, which could merit future analyses (Figure 4).

**TABLE 4:** Clinical characteristics of the total population during the basal visit (*n* = 112) and 3 months after the final visit (*n* = 89) after Andrology management. Significant differences (*p* < 0.05) are depicted in bold.

Variable	Basal (*n* = 112)	End (*n* = 89)	*p*‐value
**IIEF** (5–75 scores), basal visit	33 ± 13	50 ± 16	**<0.001**
**IIEF‐EF** (1–30 scores), basal visit	10 ± 6	21 ± 10	**<0.001**
**mSLQQ‐QOL** (0–100 scores), basal visit	27 ± 13	54 ± 24	**<0.001**
**Anxiety (HAD scale**, 0–21 scores, mean ± SD)	5 (2–8)	4 (2–9)	**0.043**
**Depression (HAD scale**, 0–21 scores, median (RIQ))	3 (1–6)	2 (0–6.5)	**0.043**

*Note*: IIEF‐EF: Index of Erectile Function questionnaire (erectile function); the modified Sexual Life Quality Questionnaire (mSLQQ‐QOL); Hospital Anxiety and Depression Scale (HAD) as Anxiety (HADA) or Depression (HADD).

## 4 DISCUSSION

The results showed that ED was present as the primary clinical manifestation in 62% of cases, with CVD occurring concurrently, with a greater ED susceptibility in eNOS 786‐TT and the aa/ac of intron 4 VNTR genotypes. Our data could merit future analyses, mostly in terms of effective therapeutic management work‐up in high‐risk CVD individuals.

This finding is consistent with the results reported by other studies in which ED was a potential independent marker for cardiovascular damage.^26^, ^27^ Clinicians must assess ED in middle‐aged men, especially those who might be at CVD risk. After andrological treatment, our patients indicated significantly improved EF and sexual quality of life with a tendency of improved cardiovascular ejection fraction related to the G894‐T polymorphism. This implies that the eNOS genotype and sexual dysfunction pose an excellent opportunity to conduct a general workup during cardiac rehabilitation programs.

One important objective of cardiac rehabilitation is to restore adequate sexual function and satisfaction. Our evidence shows that there were missed opportunities to undertake a risk assessment and to provide CVD intervention in half the ED cases.^28^ ED, as a sentinel marker and an independent marker of CVD,^29^, ^30^ could be the first clinical presentation of subclinical endothelial dysfunction disease, especially in younger men.^31^ This is important because it demonstrates that ED provides physicians with a unique opportunity to see the possible future of their patients' cardiovascular health^27^ and to make specific CVD evaluations.^32^ According to our data, men with ED should be specifically targeted for CVD prevention strategies both pharmacologically and in terms of lifestyle changes, especially for cigarette smoking and obesity. Indeed nicotine exposure increases the sympathetic nervous system tone by causing vasoconstriction and, thereby, reducing penile blood flow. It also promotes endothelial dysfunction by impairing EF. Hence the sexual function could be incorporated into patients’ medical history, taken by the physicians treating individuals with CVD,^33^ and not merely as part of the diagnostic follow‐up, but as a means to pursue tangible and essential benefits in quality of life and cardiovascular outcomes^26^ in any cardiovascular risk‐screening program.^34^ Furthermore, iPDE5 was found to be an effective, safe, and well‐tolerated treatment for ED in the present study population, as previously described.^35^, ^36^

The association between CVD and ED is greater than expected based on age. A growing body of evidence supports the use of emerging prognostic markers to further understand cardiovascular risk in men with ED, but very few markers have been prospectively evaluated.^37^ Our results indicated that the genotypes of eNOS polymorphisms and the intronic variant could be predictive of an elevated risk of ED, suggesting a need for further investigation into their impact on cardiovascular function. Moreover, a significant association of eNOS T‐786C (*p* = 0.02) and G894T (*p* = 0.005) with altered susceptibility to vasculogenic ED has been previously observed^38^ due to the G894T variant (*p* = 0.002 in a dominant model) and the T‐786C variant (*p* = 0.004 in a recessive model). Consequently, targeting vascular endothelial dysfunction through eNOS gene variants is a promising biomarker because of its potential involvement in CVD progression.^39^

In the presence of cardiovascular risk factors, endothelial dysfunction is frequently encountered and compromises the NO levels synthesized in the endothelium by the eNOS enzyme, which plays a crucial role in not only vascular homeostasis but also in regulating blood flow and blood pressure.^40^ Some reasons like decreased NO synthesis may cause circulating NOS inhibitor levels to rise, which contribute to vascular changes (e.g., higher arterial pressure) in different illnesses,^41^, ^42^ such as coronary artery disease.^43^ In this context, the eNOS G894T and T786C gene variants^20^, ^44^ dramatically reduce NO production and transcriptional activity,^45^, ^46^ which support their functional importance^47^ as ED risk pathogenesis factors.^45^, ^48^, ^49^ Nowadays, prevailing experimental and clinical data suggest that decreased NO bioavailability accelerates the progression of atherosclerosis, presumably through mechanisms like platelet activation, vascular smooth muscle proliferation, leukocyte adhesion to the endothelium, and increased vascular production of reactive oxygen species.^50^, ^51^ What is more, the presence of the eNOS 4a4a genotype represents a predisposing condition to cardiovascular events, particularly to acute myocardial infarction.^52^

A recent meta‐analysis^53^, ^54^ has shown a significant association of ED risk and G894T (GT + TT vs. GG: OR = 2.13, 95%(confidence interval (CI) = 1.08–4.19), T786C (CC vs. CT + TT: OR = 3.29, 95%CI = 2.30–4.72) in Caucasians and Asians (OR = 2.08, 95%CI = 1.53–2.84; odds ratio (OR) = 3.13, 95%CI = 1.35–7.25, respectively). In addition, the intron 4 VNTR polymorphism has been associated with ED risk in only Caucasian subjects (aa vs. bb + ab: OR = 2.38, 95%CI = 1.15–4.93). In this context, the eNOS G894T and T786C gene variants^20^, ^46^ dramatically reduce NO production and transcriptional activity,^45^, ^46^ which supports their functional importance^47^ as ED risk pathogenesis factors.^45^, ^48^, ^49^ Furthermore, genetic polymorphisms at other loci, such as replication protein A1 (RPA1) or protein disulfide isomerase (PDI), or in other eNOS complex members, would be necessary.^42^ In fact, there is some evidence to suggest that the T‐786C allele results in the increased expression of the enzyme and might, consequently, provide a protective mechanism from CVD, which suggests the presence of some essential binding sites for transcription‐enhancing proteins.^55^

## 5 LIMITATIONS

It is well‐known that observational studies can be affected by several biases. First, the sample size was limited by a “convenience sample” from a single center with a high 25% patient loss. Although this is a prospective study, only one center participated in this study, which affects the reliability of its conclusion along with the short follow‐up of patients. Moreover, geographical location, lifestyle, dietary habits, or limitations in detection methods can influence these findings and cardiovascular risk. There are also other comorbidities, such as sleep‐disordered breathing (i.e., obstructive sleep apnoea), that share a number of common risk factors and comorbid conditions, including obesity, male gender, advancing age, metabolic syndrome, and hypertension.^56^ A comprehensive analysis in future studies would be helpful. Therefore, our results should be interpreted after taking into account genetic backgrounds, the multifactorial nature of ED, and the fact that the results need to be replicated in other ethnic populations. Besides, antihypertensive drugs, depression and anxiety are the main factors to affect ED.^57^ Ejection fraction is not a marker of endothelial dysfunction on its own but is a marker of a worse prognosis of cardiovascular morbidity and mortality associated with atherosclerotic CVD in this type of patient. Therefore, we believe that knowing this relation for our study is relevant. Finally, considering that bias may have a major impact on the validity and reliability of the research findings, the following actions were taken to minimize it: 1/ standardized protocols and validated questionnaires help to ensure that observations were made consistently and objectively; 2/ blinding pharmacogenetic information to clinicians. All this ensures that observer expectations and preconceptions do not impact their observations or interpretations of facts; 3/ prospective design.

In conclusion, this study found that eNOS polymorphisms are associated with an increased risk of developing ED and more CVD male patients. ED could also improve the identification of suitable CVD patients for screening and would lead to early detection and enhance therapeutic management. Large‐scale prospective studies, including concurrent assessments of serum NO levels, could be done to fully understand the effects of eNOS polymorphism on susceptibility to CVD.

## AUTHOR CONTRIBUTIONS

Conceptualization, Ana Segura and Ana M Peiró; methodology, Ana M Peiró and Javier Muriel; software, Pau Miró; formal analysis, Javier Muriel and Pau Miró; investigation, Laura Agulló, Vicente Arrarte, and Ana M Peiró; resources, Ana Segura and Ana M Peiró; data curation, Ana Segura, Javier Muriel, and Pau Miró; writing—original draft preparation, Ana Segura, Javier Muriel, and Ana M Peiró; writing—review and editing, Thomas Zandonai and Ana M Peiró; visualization, Javier Muriel, Thomas Zandonai, and Ana M Peiró; supervision, Thomas Zandonai and Ana M Peiró; project administration, Ana M Peiró; funding acquisition, Ana Segura, Thomas Zandonai, and Ana M Peiró All authors have read and agreed to the published version of the manuscript.

## CONFLICT OF INTEREST STATEMENT

The authors declare no conflict of interest.

## ETHICS STATEMENT

The study was conducted in accordance with the Declaration of Helsinki and approved by the Ethics Committee Board of the Dr. Balmis General University Hospital of Alicante (code: PI2017‐34).

## PATIENT CONSENT STATEMENT

It was obtained from all the subjects involved in the study. Written informed consent was obtained from the patients to publish this work.

## Supporting information


**TABLE S1** Pharmacological management of the sample population.

## Data Availability

The data that support the findings of this study are available on request from the corresponding author. The data are not publicly available due to privacy or ethical restrictions.
