# Supplementary material for: Erectile dysfunction in cardiovascular patients: A prospective study of the eNOS gene T‐786C, G894T, and INTRON variable number of the tandem repeat functional interaction
Source: Andrology. 2024 Jul 1;13(4):794–803. doi: 10.1111/andr.13671 (PMC12006881; doi:10.1111/andr.13671)
Supplement: Supplementary file 1 — TABLE S1 Pharmacological management of the sample population. [file ANDR-13-794-s001.docx]

| **Pharmacological treatment**  n (%) | **Total population**  (n=112) |
| --- | --- |
| **Andrological management** |  |
| Phosphodiesterase 5 inhibitors | 85 (76) |
| Virirec | 9 (8) |
| Muse | 5 (4) |
| Intracavernous injection | 1 (1) |
| **Cardiovascular drugs** |  |
| B-blockers | 81 (72) |
| Angiotensin converting enzyme inhibitors | 36 (32) |
| Angiotensin II receptor antagonists | 41 (37) |
| Calcium channel blockers | 26 (23) |
| Acetylsalicylic acid | 95 (85) |
| Aldosterone antagonist | 5 (4) |
| Diuretic | 23 (21) |
| Anti a1 | 9 (8) |
| Anticoagulant | 14 (13) |
| Oral antidiabetic | 46 (42) |
| Insulin | 19 (17) |
| Statins | 97 (87) |
| Nitrates | 1 (1) |
| Ezetimide | 32 (29) |
| Thienopyridine | 31 (28) |
| **Other drugs** |  |
| Antidepressant | 8 (7) |
| Antipsychotic | 1 (1) |
| Opioids | 5 (4) |

**Table 1S.** Pharmacological management of the sample population
